# Feasibility Study on Additive Manufacturing of Feed Horn Operating in D-Band

**DOI:** 10.3390/s25020523

**Published:** 2025-01-17

**Authors:** Giuseppe Addamo, Lorenzo Scalcinati, Mario Zannoni, Oscar Antonio Peverini, Flaviana Calignano

**Affiliations:** 1 Consiglio Nazionale delle Ricerche, IEIIT, Corso Duca degli Abruzzi 24, 10129 Torino, Italy; 2 Physics Department, University of Milano Bicocca, Piazza della Scienza 3, 20126 Milano, Italy; l.scalcinati@campus.unimib.it (L.S.);; 3 Department of Management and Production Engineering (DIGEP), Politecnico di Torino, Corso Duca degli Abruzzi 24, 10129 Torino, Italy; flaviana.calignano@polito.it

**Keywords:** PBF-LB/M, additive manufacturing, smooth wall horn, D-band

## Abstract

This paper presents the outcomes of a feasibility study on the manufacturing of D-band horn antennas through the Low Power Bed Fusion process. Different prototypes have been realized and tested, showing nice results in terms of the co-polarization component. On the other hand, a spurious cross-polarization component is present in the radiation pattern even in the principal planes, limiting the device to single-polarization applications. A mechanical study of the realized devices has been conducted on the horn internal channel to understand the reasons for this issue, and, subsequently, to address where to improve the manufacturing process.

## 1. Introduction

The scientific community is showing, nowadays, an increasing interest in the sub-terahertz frequency band for climate monitoring and astrophysical observation [1,2]. Sub-terahertz remote sensing offers a unique capability for improving climate-change and weather monitoring from space thanks to its higher sensitivity to fundamental observable parameters, such as columnar water vapor distribution. This parameter is an indicator of a proxy of extreme phenomena that often hit the Mediterranean countries. On the other hand, astrophysical research increasingly quests for the realization of focal planes populated by receivers with tens or even hundreds of feed horns. The QUBIC (Q&U Bolometric Interferometer for Cosmology) observation mission is a significant example. The QUBIC experiment is based on bolometric interferometry in order to measure the B-mode polarization anisotropies of the Cosmic Microwave Background (CMB). The experiment consists of ground-based observations of the sky in two main spectral bands centered at 150 and 220 GHz [3]. The instrument, which is already operative in a reduced version with 64 horns, known as TD (Technological Demonstrator), will be composed by a rotating half-wave plate and a polarization grid exploited to modulate the measured polarization of the incoming signal from the sky. In the final version, an array of 400 corrugated horns collects the radiation and subsequently suitably detects it.

In both cited application domains, for the horns’ realization, electroforming [4] represents, nowadays, the best manufacturing technique to guarantee a good accordance between simulated and measured performance in terms of both radiation pattern and insertion loss. Its cost is the main drawback of this technique that, indeed, prevents its use for horn arrays larger than a few elements.

An affordable alternative, already implemented in radio-astronomical instrumentation, is the metallic platelet technique [5]. This approach makes use of packs of metal sheets (platelets) holed to reproduce the feed horn profile [6,7,8,9]. Unfortunately, at frequencies higher than ~100 GHz, even the metallic platelets show some limitations due to the discontinuities/air gaps among the plates that can compromise electromagnetic and thermal performances. It is worth mentioning that the front ends of these instruments are, in most cases, cooled at cryogenic temperatures. Therefore, this drawback is particularly critical for the study of the Cosmic Microwave Background, where the control of the instrumental systematic effects is fundamental in the polarized B-modes’ detection [10,11,12].

A viable solution to the cited problems can be represented by Additive Manufacturing (AM) processes [13], whose employment in the microwave/antenna community has increasing exponentially in the last ten years thanks to their features such as net shapes and free-form capabilities. An interesting overview on the advantages of these and the other AM features is reported in Ref. [14].

In the case of all metal devices, such as horn antennas and relevant feed-chains, the most suitable process is Low Power Bed Fusion with an aluminum-based alloy (PBF-LB/M/AlSi10Mg). Its usual application domain is in the realization of devices operating in K/Ka bands (i.e., from 10 to 30 GHz) [15,16,17]. The manufacturing of complex waveguide channels at a higher frequency, indeed, suffers on some process limits in terms of accuracy and surface roughness. However, considering the manufacturing of horn antennas, quite interesting examples can be found even at higher frequencies (such as in Q/V and W bands [18,19,20,21,22]). In the cited papers, different horn geometries have been considered, from the classical conical one [19], to a corrugated solution [21] and smooth wall architectures [22].

Stereo-lithography is a quite interesting option in the panorama of AM processes thanks to its superior surface finishing and accuracy, but the metallization of the inner channels is, in general, not an easy task [23,24,25]. Binder Jetting [26] or Fused Deposition modeling (FDM) [27] have also been employed in the realization of horn antennas, thanks to the lower-cost production, but, in general, its performances are worse.

Starting from the nice outcomes from Ref. [22] in terms of measured cross-polarization and return loss, here, we discuss the results of a feasibility study on the applicability of a PBF-LB/M process to manufacture feed horns in the D band (110–170 GHz). In particular, as a significant benchmark, we have considered the design of a feed horn operating in the frequency range of 130–160 GHz with radiation performance compatible with the feed horn of the QUBIC experiment.

We have manufactured and fully electromagnetic characterized three AM prototypes with different external profiles. Despite the high-frequency operative band, we have observed a nice accordance with the predicted performance in the co-polar component. The radiation patterns present, unfortunately, an undesired cross-polarization term, putting in evidence that the actual technological readiness of this manufacturing process is not sufficient for the realization of a dual-polarization system in the D-band. Through a mechanical analysis of the realized part and elaboration of the relevant data, we have focused on the issues that can have generated the measured problem. These outcomes can be used as starting points to improve the manufacturing process.

The article is divided in the following way. Section 2 deals with the choice of the horn architecture and its design. Section 3 discusses the manufacturing of the prototypes and the antenna measurements. Finally, Section 4 is devoted to mechanical nondestructive testing, through tomography, and the relevant data elaboration.

## 2. Feed Horn Design

**Figure 1:**
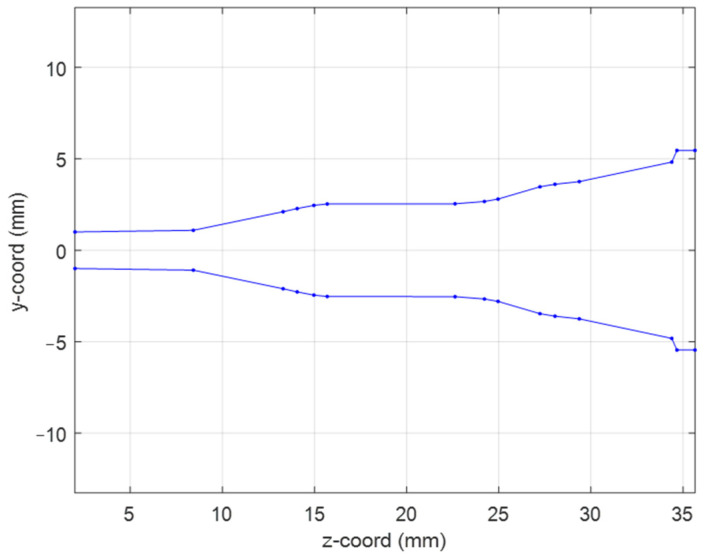
Geometry of the designed feed horn.

**Figure 2:**
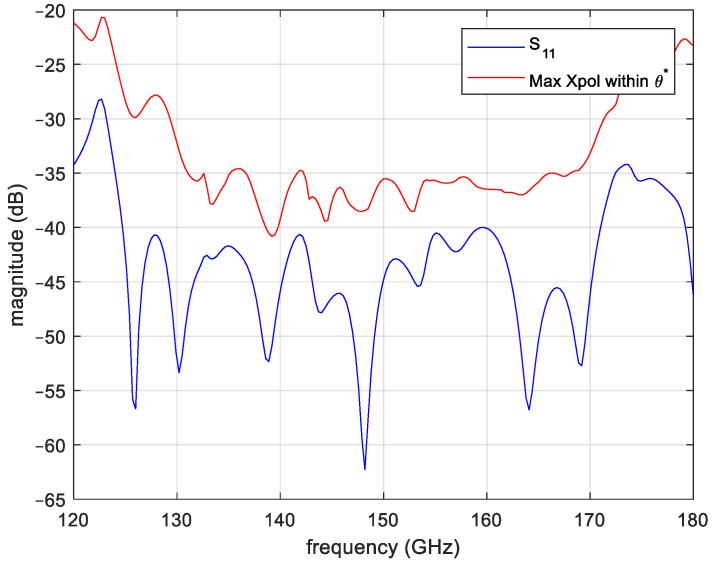
Simulated performance of the smooth wall horn shown in Figure 1.

In the present study, we consider the requirements of QUBIC feed in terms of its operative band, return loss, cross-polarization level, and field taper at the reflector illumination angle. Although corrugated horn architecture is the most suitable in terms of cross-polarization performance, its manufacturing through the AM process is particularly cumbersome, since the internal corrugations are not self-supporting, and the device can be realized only considering an inclination of the part in the building machine. This usually translates into worse performance of the antenna. On the other hand, smooth wall solutions do not present this problem since their structure is fully self-supporting, so the part can be oriented in the building machine parallel to the laser axis assuring better manufacturing accuracy [22]. For this reason, we have adopted this architecture in the present study. The input and radiating circular waveguide diameters have been selected identically to the QUBIC horn i.e., $D_{i}$ = 2 mm and $D_{ext}$ = 10.92 mm, respectively. According to the design technique presented in Ref. [28], we have obtained the horn geometry by optimizing its generatrix profile with 14 control nodes. We have chosen such high value to cover the large (around 21%) operative bandwidth, i.e., from 130 GHz to 160 GHz. Figure 1 reports a 2D section of the final horn profile. According to the reference system shown in the plot, the radial and longitudinal coordinates of the horn generatrix are (in millimeters):

$\rho_{i}$ = {1 1.09 2.107 2.279 2.454 2.531 2.542 2.667 2.798 3.470 3.608 3.755 4.824 5.46 5.46};

$z_{i}$ = {0 6.42 11.3 12.06 12.97 13.69 20.63 22.22 22.96 25.23 26.05 27.37 32.39 32.67 33.65};

Figure 2 reports the simulated performance of the horn in terms of return loss and maximum cross-polarization level within the angle θ*=60°. The plot shows a quite nice level of return loss and cross-polarization, both better than −35 dB in a wider frequency band. The design has been carried out using an in-house numerical code. This code is based on the mode-matching and Coupled Integral Equation Technique (CIET) [29,30]. To this end, first, the horn profile is discretized using a discretization step equal to λ/30, where λ is the free-space wavelength at the highest operative frequency (i.e., 160 GHz). The profile staircase approximation is, then, interpreted as a cascade of step discontinuities in circular waveguide. The electromagnetic behavior of the antenna is computed applying the Equivalence Theorem, i.e., closing each discontinuity with a perfect electric conductor and introducing unknown magnetic equivalent currents. A system of coupled integral equations is then formally obtained by relating the unknown equivalent current on the i-th discontinuity with the relevant currents on the (i − 1)-th and (i + 1)-th discontinuities. By expanding these currents on the basis of the apertures modes, this system is converted into a tridiagonal-blocks system that is effectively solved by using the Gauss elimination method.

**Figure 3:**
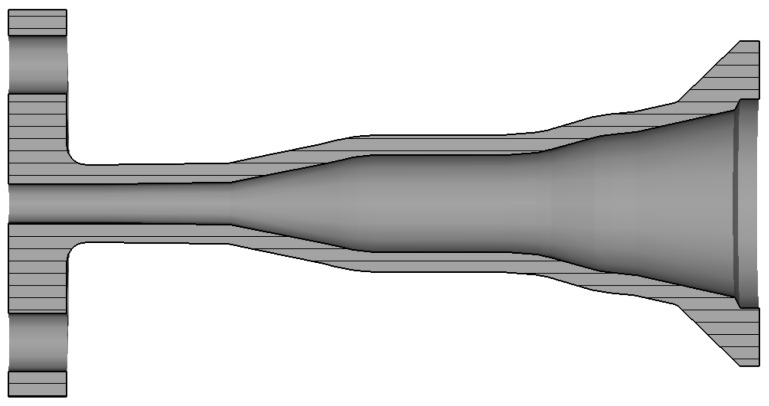
CAD model of the smooth wall horn of Figure 1.

The horn external profile (shown in Figure 3) has been designed to minimize the envelope but at the same time to guarantee sufficient rigidity of the antenna. According to previous experience in the manufacturing of feed horns [22], we have employed a constant metal thickness of 1 mm along the central part of the antenna following the internal profile. The radiating aperture presents instead a corona of around 17 mm diameter to reduce the effect of the radiation of the external currents. The connection between the central and radiating external parts has been realized by a smooth conical external transition. Finally, the input circular waveguide has a larger cross-section, with holes for pins and screws, for the connection of the horn to the measurement setup. An input circular waveguide length of 5 mm has been included to simplify the mounting with the measurement setup.

## 3. Antenna Manufacturing and Testing

**Figure 4:**
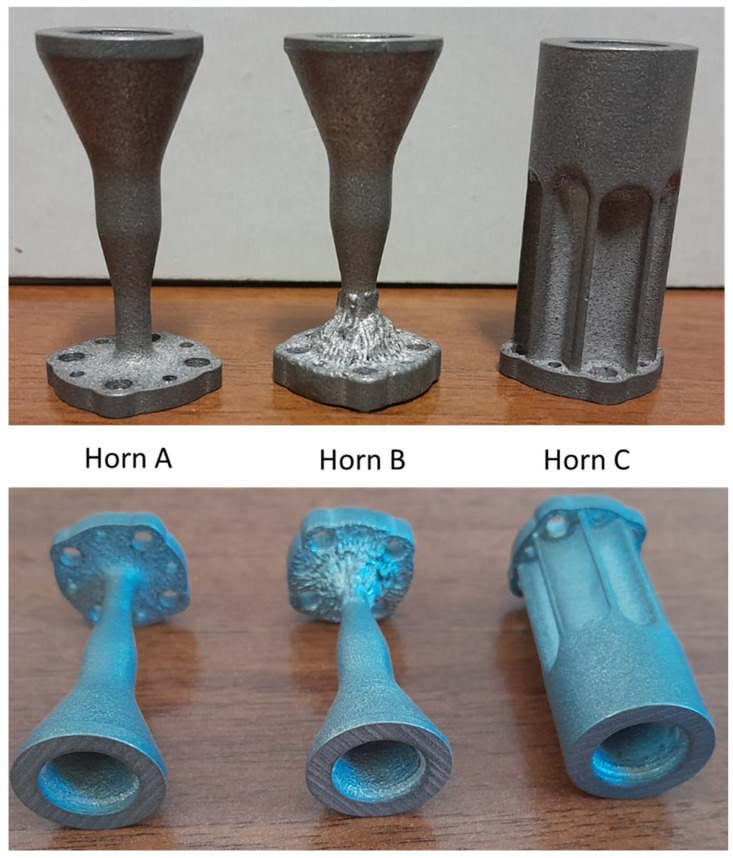
Pictures of the realized feed horns prototypes in PBF-LB/M/AlSi10Mg.

As discussed in the introduction, our intention is to understand the feasibility of PBF-LB/M process to realize microwave devices, and, in particular, feed horns, operating in the D-band. Using the EOSINT M270 Dual Mode, three prototypes have been manufactured exploiting the classical AlSi10Mg alloy. Figure 4 reports a picture of the three antennas. From the CAD model shown in Figure 3, these horns present some differences from a mechanical point of view. The first two (horn A and horn B), indeed, are quite similar to the CAD model, except for a small difference related to a conic loft in the connection region between the input flanges and the central body of the horn. We have applied this expedient to guarantee more robustness to the part. The almost conical shape of a smooth-wall horn permits, indeed, quite a neat shape solution, but some trade-offs on the external shape are necessary to avoid warp/collapse during the manufacturing and post-processing procedure. In the case of Horn C, we have considered a bulky solution to understand if it could improve the manufacturing quality of the part. We have applied a stress relieving heat treatment of 2 h at 300 deg for all the prototypes as well as a shot peening with glass microsphere using a pressure of six bar [31]. This is an essential step to reduce the roughness (from around 20 µm to around 3–8 µm) that translates into better conductivity of the internal channels and, therefore, lower metallic loss.

The measurement setup for the electromagnetic characterization of the feed horns is composed by a Vector Network Analyzer (VNA) (PNA-X N5245B) connected to a commercial transition from square to circular waveguide where the Antenna Under Test (AUT) is joined. For the beam pattern measurement, a launcher antenna (RPG Corrugated Feed Horn WR6 Type FH-CG-140) is mounted on an optical bench and connected to the VNA. Absorber material (ECCOSORB HR-10) is used to cover all the possible reflecting surfaces in the lab.

**Figure 5:**
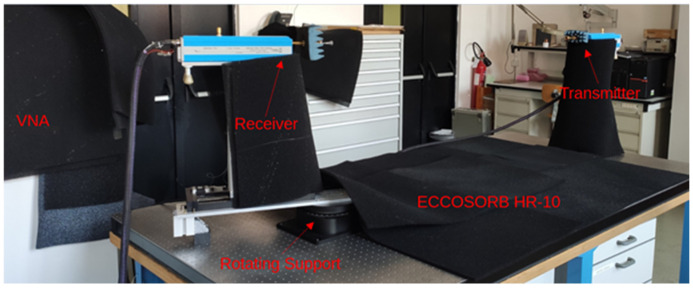
Picture of the measurement setup.

Both the launcher and the AUT are placed on proper support to guarantee the far field approximation and the reciprocal alignment in the case of boresight direction. In particular, the launcher has fixed support with a lab-jack. The AUT is mounted on a rotating platform that allows us a proper beam pattern characterization for each incidence angle. The propagation distance from the launcher aperture to the receiver aperture is 147 cm, that is much more of the Rayleigh distance ($2D^{2}/\;\lambda$) at 160 GHz, to ensure an almost plane illumination of the AUT in the operative bandwidth. We have used waveguide twists to perform measurements on the different planes, compensating their presence using straight waveguide if necessary. Figure 5 reports a picture of the overall measurement setup.

**Figure 6:**
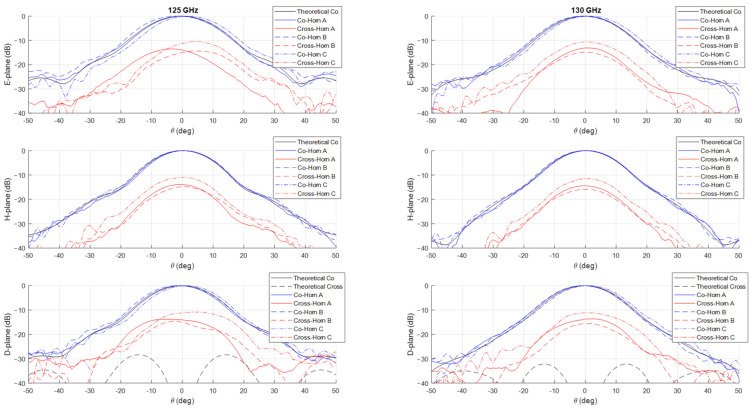
Comparison between theoretical (in black) and measured (in blue and red) co-polar and cross-polar normalized radiation patterns in the E-, H-, and D-planes at 125 GHz (**left**) and 130 GHz (**right**). The plot reports the measurements on the three prototypes Horn A (continuous lines), Horn B (dashed lines) and Horn C (dashed dot lines).

**Figure 7:**
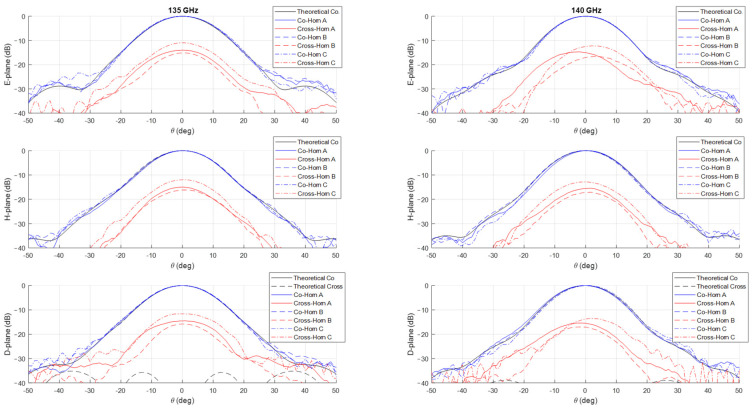
Comparison between theoretical (in black) and measured (in blue and red) co-polar and cross-polar normalized radiation patterns in the E-, H-, and D-planes at 135 GHz (**left**) and 140 GHz (**right**). The plot reports the measurements on the three prototypes Horn A (continuous lines), Horn B (dashed lines) and Horn C (dashed dot lines).

**Figure 8:**
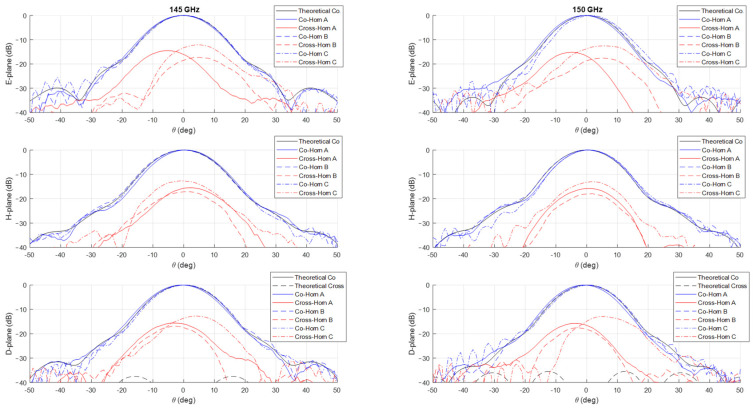
Comparison between theoretical (in black) and measured (in blue and red) co-polar and cross-polar normalized radiation patterns in the E-, H-, and D-planes at 145 GHz (**left**) and 150 GHz (**right**). The plot reports the measurements on the three prototypes Horn A (continuous lines), Horn B (dashed lines) and Horn C (dashed dot lines).

**Figure 9:**
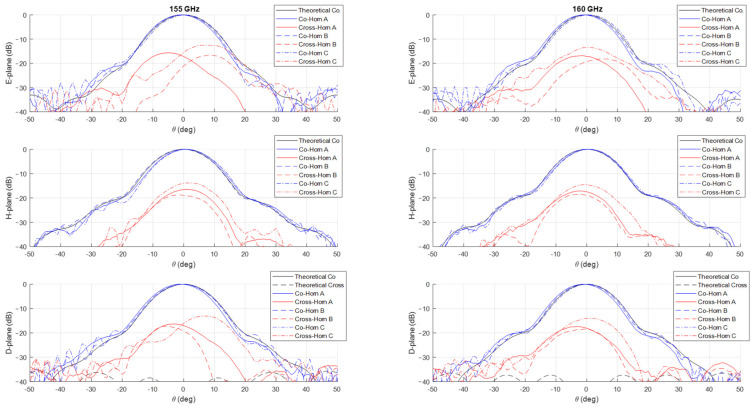
Comparison between theoretical (in black) and measured (in blue and red) co-polar and cross-polar normalized radiation patterns in the E-, H-, and D-planes at 155 GHz (**left**) and 160 GHz (**right**). The plot reports the measurements on the three prototypes Horn A (continuous lines), Horn B (dashed lines) and Horn C (dashed dot lines).

The measurements of the normalized co-polar radiation patterns in the E, H, and D planes for the three prototypes are shown in Figure 6, Figure 7, Figure 8 and Figure 9, considering a 5 GHz step in the frequency range 125–160 GHz. In all the plots, the theoretical radiation pattern is reported, showing a very good accordance between simulations and measurements. In particular, the plots show an excellent fitting with the predicted one up to the angular range $\pm 40^{{}^{\circ}}$; worse results can be observed at larger angles. This can be partially attributable to the accuracy of the measurement setup in terms of spurious reflections.

Unfortunately, as shown in the figures, all the three prototypes show a spurious cross-polarization level in the radiation pattern even on the principal planes where it should be nominally zero. This behavior is asymmetric in θ and not uniform in frequency with worse performances at lower ones. This cross-polarization component is surely the result of a spurious modal combination at the horn aperture. This combination should be composed by both circular waveguide modes with azimuthal index m≠1 and those ($TE_{1n}$ and $TM_{1n}$) related to the horizontal polarization of $TE_{11}$ mode. Note that the rectangular to circular transition is connected to the horn to excite, namely, only the vertical polarization of the$\;TE_{11}$ mode. The modes with azimuthal index m≠1 can be excited if the perfect azimuthal symmetry of the horn is not maintained along the structure. These modes are below cut-off at the input waveguide, but, as the diameter increases, gradually become above cut-off and can propagate, if excited. The modes related to the other polarization of the $TE_{11}$ mode, i.e., the horizontal one, can be present along the structure for different reasons. The first one is the imperfect connection between the horn and the transition from the rectangular and circular waveguide due to small backslash in both the screws and pins in the realized horn flange. The same polarization purity in transmission of the rectangular to circular transition can play a role on this problem by exciting a non-negligible component of the horizontal ${TE}_{11}$ mode. Finally, another possible concause is the imperfect concentricity of the different cross-sections along the internal channel. The verification of the first two hypotheses is quite cumbersome, while it is possible to control the last point through a mechanical nondestructive analysis on the prototypes. We discuss the details and results of this investigation in the next section.

We have measured the metallic losses of the three horn prototypes by closing the radiating aperture with a metallic sheet and measuring the reflection coefficient at the horn input port. The mean measured losses are around 0.3 dB compatible with an equivalent surface resistivity of 16–18 µΩcm. As proved in different published articles [32,33], A silver plating can be applied, if necessary, to improve this value.

**Figure 10:**
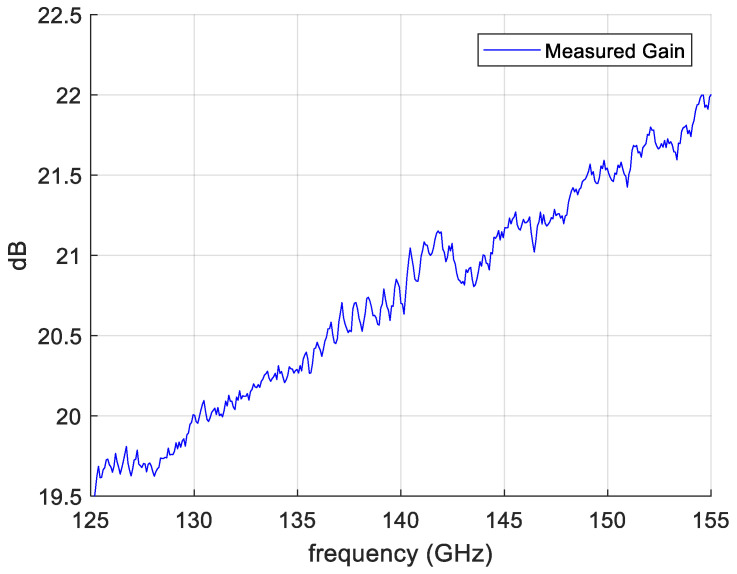
Measured gain of Horn B.

Figure 10 reports the measured gain of horn B as a function of the frequency. We have observed a similar behavior for the other horns. Finally, the measured reflection coefficient of the horn connected to the rectangular to circular transition is lower than −27 dB in all the frequency range 130–160 GHz.

## 4. Mechanical Measurements of the Horn Prototype

We have carried out a deep investigation for better understanding the reason for the measured cross-polarization levels. To this end, we have preferred a non-invasive inspection through tomographic analysis on the horns using the instrument GE Phoenix v|tome|x s. In particular, we have applied this inspection on horn A and B. In the following, we report the analysis for horn B, since similar results and conclusions were maintained for horn A.

**Figure 11:**
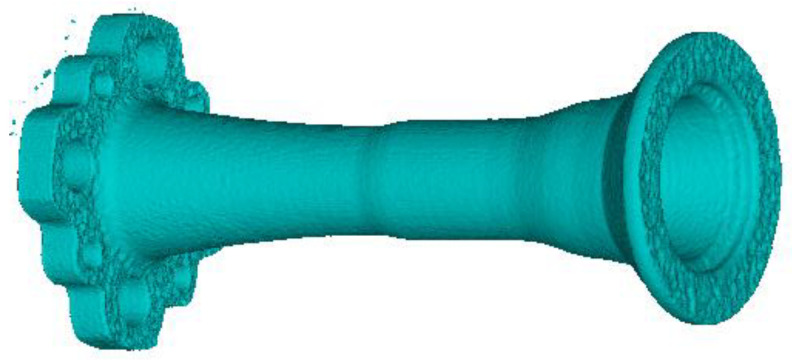
Result of the tomography on the horn prototype B.

During the acquisition, the tomographic scanner has acquired a set of around 470,000 3D-points. Figure 11 shows the 3D model generated from this acquired data.

Starting from this dataset, the idea is to re-construct, section by section, the realized internal channel of the horn and then compare these sections with the nominal ones. In order to maintain the highest flexibility and generality, we have exploited a numerical algorithm to deduce, for each section of the internal channel, the best fitting ellipse Υ. Since the data are not uniform along the longitudinal *z*-axis of the horn, a discretization in steps of 0.05 mm have been applied. Note that, as is well known, at least six points are necessary to define an ellipse in a plane.

**Figure 12:**
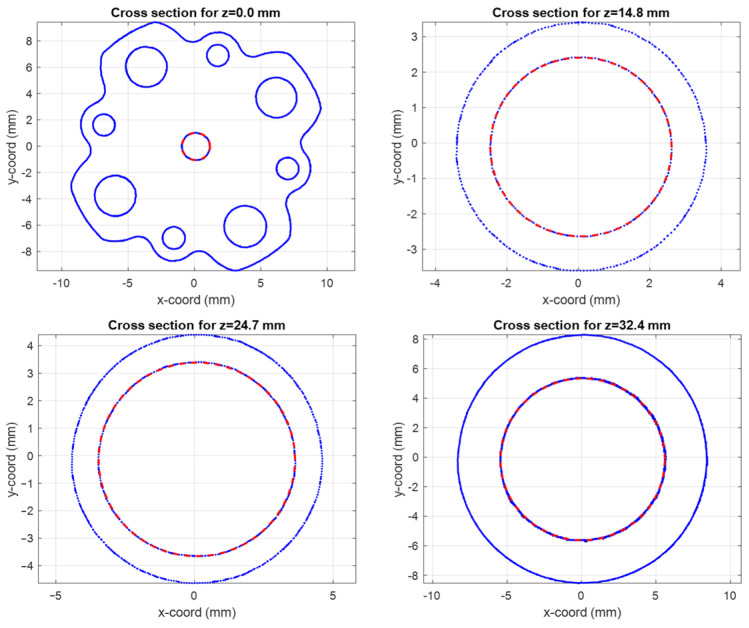
Cross-sections of the horn at different longitudinal coordinate z (input section: **top left**, central sections: **top right** and **bottom left**, close to the radiating aperture: **bottom right**). The blue points refer to the tomographic data, the red ones to the ellipse best fitting Υ.

**Figure 13:**
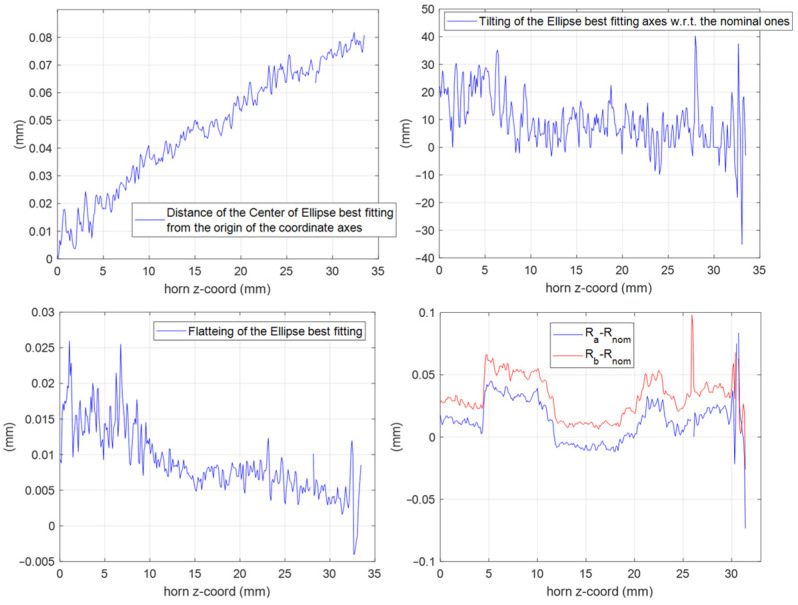
Results of the elaboration of the tomographic data in terms of ellipse best fitting Υ along the horn sections. **Top left**: deviation of the center position. **Top right**: tilting angle of the ellipse main axes with respect to the nominal one. **Bottom left**: flattening of the ellipse. **Bottom right**: differences between the major ($R_{a}$) and minor ($R_{b}$) semi-axes of the ellipse and the nominal radius ($R_{nom}$) for all the horn sections.

The effectiveness of this numerical reconstruction is shown in Figure 12 where the comparison between the acquired points (in blue) and the best fitting ellipse Υ (in red) is reported, as an example, in four z-sections of the horn: the input one, two centrals, and one close to the radiating aperture. The good fitting shown in the plots confirms the choice of this mathematical model. From the analytical expression of Υ, we have derived, for each longitudinal section, the center position and the major $\left(R_{a}\right)$ and minor $\left(R_{b}\right)$ semi-axes of Υ as well as the tilt of the main directions of the ellipse with respect a fixed (x,y) reference system centered at the horn input waveguide. A summary of this analysis is reported in Figure 13. In the plot is evident a deviation of the centers of the various Υ with respect to the longitudinal axis of the horn leading to an overall drift of around 0.1 mm along the structure. At the same time, a non-negligible tilting of the main axes is present. These two aspects represent surely a significant concause of the measured cross-polarization component on the radiation patterns reported in Figure 6, Figure 7, Figure 8 and Figure 9. As far as the shape of the cross-section is concerned, the analysis shows a good circularity with semi-axes $R_{a}$ and $R_{b}$ quite close (with a difference almost everywhere lower than 0.05 mm) to the nominal radius $R_{nom}\;$except in the radiating region where it is around 0.07 mm.

## 5. Conclusions

Despite the not excellent agreement with the desired results in terms of the cross-polarization component, the nice outcome in the measured co-polar component proves that PBF-LB/M is an interesting manufacturing technique still in the D-band, and it can be effectively employed for the manufacturing of single linear-polarization devices. The mechanical analysis of the horn shows the necessity of better control, in the manufacturing process, over the absolute center position of the cross-section to reduce the observed drift. Future work will focus on both improving the manufacturing in this aspect and reducing the electromagnetic noise in the measurement setup for better characterizing these antennas in a wider angular region. Finally, the realization of an integrated version of the horn with an orthomode transducer can be a future step to minimize the uncertainty in the multimodal connection of the horn with the transition from a rectangular to a circular waveguide.

## Data Availability

Data will be made available on request.
